# Allergy to pets and new allergies to uncommon pets 

**DOI:** 10.5414/ALX01842E

**Published:** 2017-08-04

**Authors:** M. Curin, C. Hilger

**Affiliations:** 1 Division of Immunopathology, Department of Pathophysiology and Allergy Research, Center for Pathophysiology, Infectiology and Immunology, Medical University of Vienna, Austria and; 2 Department of Infection and Immunity, Luxembourg Institute of Health, Esch-sur-Alzette, Luxembourg

**Keywords:** allergy to pets, animal allergens, furry animals, cat, dog, rabbit, guinea pig, hamster, exotic pets, component-resolved diagnosis

## Abstract

Abstract. Animal dander is an important source of respiratory allergens, and sensitization to allergens from cat and/or dog during childhood represents a risk factor for the development of asthma and rhinitis later in life. The identification and characterization of allergenic components is crucial to improve diagnosis and therapy in patients with allergy to pets. Allergens from furry animals belong to a restricted number of protein families, a large majority are lipocalins or albumins, some are secretoglobins or latherins. Animal dander contains cross-reactive molecules and current efforts aim at defining species-specific allergens that have a high diagnostic sensitivity. Component-resolved diagnosis allows to discriminate genuine sensitization from cross-sensitization. This review contains a detailed description of allergenic components of cat, dog, horse, and small mammalian pets. Sensitizations to exotic pets, a newly emerging issue, are also discussed.

German version published in Allergologie, Vol. 39, No. 6/2016, pp. 247-255

## Introduction 

Pets, in particular mammalian furry pets, are an important source of indoor allergens. Sensitization to animal allergens is considered a major risk factor for development of asthma and rhinitis [36]. In Europe and the US, pets are very popular: depending on the region, 30 – 60% of all households have one or more domestic animals. The most common pet animals are dogs and cats, followed by fish, small mammals, birds, and exotic animals. Exposure is high in houses with pets and occupational environment, but passive transfer to public places has been shown for major cat, dog, and horse allergens [63]. Cat and dog allergens are well characterized molecules whereas allergens from small mammals and uncommon pets are less well known. 

## Cat 

Prevalence of cat allergy is around 20% within atopic population, and sensitization to cat dander represents a strong risk factor for asthma [36]. Cat allergens were detected in households with and without cats, in schools, means of public transportation, and other public places since they adhere to clothes and can stay airborne for a long time [63]. Cat allergic patients showed IgE reactivity with proteins from hair, dander, skin, saliva, serum, and urine [63]. The best studied animal allergen Fel d 1 is recognized by more than 90% of cat allergic patients, accounts for 50 – 90% of the total allergenic activity in cat dander, and is a member of the secretoglobin protein family [36]. Due to the high prevalence of patients’ recognition of Fel d 1 and species specificity, Fel d 1 is considered to be a reliable marker allergen for cat allergy. The usefulness of Fel d 1 to predict asthma development in children was tested by Gronlund et al. [23] where the authors found that IgE levels against Fel d 1 were significantly higher among children with asthma compared to children with rhinoconjunctivitis. A recent study demonstrated that sensitization to Fel d 1 in childhood was significantly associated with allergic symptoms to cat at age 16 years [4]. Another cat allergen, serum albumin (Feld 2), was identified as a minor but highly cross-reactive allergen between mammals [58]. Fel d 2 was recognized preferentially by cat-allergic patients suffering from severe manifestations of atopy (e.g., atopic dermatitis, allergic asthma) [52, 62]. Furthermore, sensitization to Fel d 2 is also responsible for pork-cat syndrome where IgE antibodies originally directed against cat serum albumin cross-react with porcine albumin and this can lead to severe systemic reactions in some patients upon consumption of medium cooked pork or raw meat such as sausages or ham [29, 50]. A second major cat allergen, Fel d 4 was the first lipocalin allergen that was isolated from cat and 63% of cat-allergic subjects exhibited IgE reactivity to Fel d 4 but typically at lower levels than to Fel d 1 [56]. Two immunoglobulins, IgA and IgM, were also identified as cat allergens and they are referred as Fel d 5 and Fel d 6 respectively [1, 2]. Interestingly, galactose-α-1,3-galactose seems to be a major IgE-binding epitope of these two cat allergens [22] and in general, this carbohydrate epitope is responsible for delayed meat allergy [11, 26]. However, the exact crosslink with cat allergy is still unclear [3]. Two minor cat allergens, Fel d 7 and Fel d 8, lately have been added to the list of cat allergens [57]. A detailed overview of cat allergens is shown in Table 1. 

## Dog 

Dogs are kept as household pets worldwide, but dog allergens were also found, similar like cat allergens, in households without dogs, in schools, and other public places [63]. A large population-based study in Germany involving children and adolescents reported an IgE-sensitization prevalence of 9.7% to dog dander [55]. Dog-allergic patients reacted with more than 10 different proteins in the extracts of hair, dander, urine, skin, saliva, and serum [49, 59]. The major dog allergen Can f 1 is a lipocalin with IgE prevalence ranging from 49 – 75% depending on the studied population [13, 35, 42]. Recently, it was shown that sensitization to Can f 1 in childhood has a positive predictive value for the development of allergy in adolescence [4]. Can f 2 and Can f 4 are minor dog lipocalin allergens of less clinical importance and they show some cross-reactivity with allergens from cat and cow, respectively [35, 40, 41]. Can f 3 is a serum albumin and represents a broadly cross-reactive allergen with IgE prevalence of up to 30% [47]. Can f 5, a member of the kallikrein family, is the second major dog allergen which bound IgE antibodies from 70% of dog allergic patients [42]. This allergen was found in dog urine and no cross-reactive allergens were found in other animals. However, Can f 5 was shown to be cross-reactive with human prostate-specific antigen (PSA) and this cross-reactivity is likely responsible for human seminal plasma allergy in some female patients [6, 42]. Another member of the lipocalin family, Can f 6, was added to the list of dog allergens and its cross-reactivity with the major cat allergen Fel d 4 and horse allergen Equ c 1 can partially explain co-sensitization to several animal danders in some patients [31, 34, 45]. A detailed overview of dog allergens is shown in Table 1. 

## Horse 

Allergy to horses usually occurs among people who regularly handle horses, either professionally or for recreational purposes [63]. Nevertheless, a recent Italian study in the urban atopic population found that 5% of patients had positive skin prick test to horse dander although more than half of sensitized patients denied ever having contact with horses [38]. High levels of horse allergens were detected in classrooms and it is also likely that people carry horse dander to their homes on their shoes and clothes [63]. The major horse allergen, Equ c 1, is a member of the lipocalin family [21] with demonstrated cross-reactivity with Can f 6 and Fel d 4 from dog and cat, respectively. Sensitization to Equ c 1 has been associated with severe asthma in Swedish children [37]. Another lipocalin allergen, Equ c 2, and a highly cross-reactive horse albumin, Equ c 3, were also described but likely play a minor role in horse allergy [8, 9]. Equ c 4, a member of the latherin family is the second major horse allergen but its importance still needs to be determined [43]. Additional information on exposure rates and allergy to horses related to the occupational environment can be found in the chapter on “Occupational allergies to animals in farming environments” in this issue. 

## Rabbit 

Rabbits, formerly raised for their fur and meat, have become increasingly popular as pets. In Europe and US, they rank third after cats and dogs [64]. Saliva has been shown to be the most potent allergen source besides fur and urine [5, 51]. N-terminal sequences of two allergens, Ory c 1 and Ory c 2, have been determined and the proteins were assigned to the lipocalin family [5]. Ory c 3, a secretoglobin with high structural homology to Fel d 1, has been isolated from rabbit hair [27]. In a group of patients sensitized to rabbit, IgE prevalence to Ory c 3 was 77%. Patients reported asthma, rhinitis, and conjunctivitis upon contact with rabbits. Despite a high structural similarity, sequence identity between Ory c 3 and Fel d 1 is low, and no cross-reactivity has been observed. Ory c 3 has been detected in settled dust collected from households with rabbit pets. Another lipocalin allergen, Ory c 4, has been isolated from rabbit fur. It has a high sequence identity to Fel d 4 and Can f 6, and IgE-cross-reactivity is highly probable [28]. 

## Guinea pig 

Guinea pigs rank forth on the scale of the most popular mammalian pets [64]. As for other animal allergens, guinea pig allergens were found in saliva, urine, and on fur. Cav p 1 is not completely characterized, but seems to be a lipocalin [16]. Two other lipocalins, Cav p 2 and Cav p 3, were isolated from lachrymal and salivary gland, respectively [32]. Both are major allergens with IgE prevalences of 65 and 54% respectively. As no cross-reactivity has been observed with cat and dog extracts, they seem to be species-specific marker allergens. Cav p 4 is a cross-reactive serum albumin, and Cav p 6 is a lipocalin with high sequence identities with Fel d 4, Equ c 1, and Can f 6 [30]. 

## Hamster 

The prevalence of hamsters in households is not known, but they seem to be less common than rabbits or guinea pigs. Nevertheless, there are a number of case reports on anaphylactic reactions following hamster bites and on asthmatic symptoms following exposure to these animals [48]. Hamsters are not a uniform animal group, but they are subdivided into different species. 

The most popular hamster species are the Golden or Syrian hamster (Mesocricetus auratus), and the two dwarf hamsters, namely the Roborovski hamster (Phodopus roborovskii) and the Djungarian or Siberian hamster (Phodopus sungorus). The European hamster (Cricetus cricetus) is mainly used in animal experimentation. Allergens from these different species are not identical. Patients allergic to the Siberian hamster did not react to commercial skin prick test solutions made from the European or Golden hamster [25]. The major allergen of the Djungarian hamster has been identified as lipocalin [60]. The protein showed some cross-reactivity with Roborovski hamster, however no cross-reactivity was observed with Golden or European hamster. The major allergen, Mes a 1, of the Golden hamster has been identified recently as male-specific submaxillary gland protein and it was found to be different from the major allergens of the two Phodopus hamsters [25]. This is particularly relevant, as conventional skin test solutions are all derived from Golden hamster or European hamster. 

## Uncommon pets 

Rabbits, guinea pigs, and hamsters are often classified as uncommon pets. However, these pets have gained much popularity, and allergens are already well characterized, therefore they have been described in separate paragraphs above. Mouse and rat allergens have been the topic of a special issue on occupational animal alergens (Allergologie. 2016; *39:* 86-95). 

There are an increasing number of case reports on allergic reactions to exotic pets such as chinchillas, gerbils, ferrets, pigs, monkeys, spiders, amphibians, and reptiles such as lizards and chameleons [15]. 

All animals can potentially cause hypersensitivity reactions. Certain animals such as snakes, lizards, and spiders can in addition cause signs of envenomation that have to be differentiated from a hypersensitivity reaction [48]. Respiratory symptoms are the most commonly reported manifestations; anaphylactic shock has been reported upon animal bites. Diagnosis is often difficult as commercial extracts for skin prick tests and IgE diagnosis are lacking for a number of exotic animals. In some cases, symptoms may be caused by the food given to the animal such as seeds, nuts, insect larvae, or dried fish or shrimps. 

For most exotic pets, only isolated case reports have been published, and allergens have not yet been identified [48]. For ferret, a pet with increasing popularity in the US, several IgE-binding proteins have been described in urine [20]. A 17-kDa protein was isolated and partially characterized by MS/MS, but the protein identity could not be determined. A patient reported asthma since the acquisition of two prairie-dogs and aggravation of symptoms when handling the bedding material. Two putative lipocalins were identified in the prairie-dog feces particles that were contaminated with urine [18]. Another patient reported respiratory and cutaneous symptoms upon exposure to a chinchilla pet [17]. Identified IgE-binding proteins in fur extract were a lipocalin and a protein kinase inhibitor. Systemic reactions have been reported upon bites of gerbils [48]. In a case of occupational asthma to gerbil, a putative lipocalin has been identified in gerbil urine and epithelium [14]. 

Furry animals are well known to elicit allergic symptoms, but people are often not aware that spiny or scaly animals can also be a source of allergens. Two recent publications referred to patients with respiratory symptoms or urticaria upon exposure to African pygmy hedgehogs. IgE-binding proteins could be detected in dander and spines [46] as well as feces balls [19]. Several cases of asthma, rhinitis, and conjunctivitis have been described upon exposure to iguana [48]. IgE-binding proteins of 40 and 50 kDa could be detected in iguana scale extract [54]. 

## Animal allergens belong to few protein families 

Around 50% of known animal allergens belong to the lipocalin family [30]. Lipocalins are small extracellular proteins with biological functions predominantly related to the transport of small hydrophobic ligands, such as vitamins and pheromones, and are mostly produced in liver and secretory glands. Sequence identity between lipocalins is usually low but they share some conserved sequence motifs and similar three-dimensional structure of β-barrel with an internal cavity that could serve for the binding and transport of small hydrophobic ligands [24]. However, some of the lipocalins have higher sequence identity (above 50%) and show certain level of cross-reactivity at the IgE level (e.g., Fel d 4, Can f 6, and Equ c 1). Another interesting group of animal allergens are serum albumins which are the major components in the circulatory system of animals and humans. The sequences and three-dimensional structures of albumins are evolutionary highly conserved [10]. The third important protein family, the secretoglobins, includes two major allergens: Fel d 1 from cat and the recently described rabbit allergen Ory c 3. Despite of structural similarity, no cross-reactivity at the IgE level could be observed between Fel d 1 and Ory c 3 [27]. Secretoglobin allergens are mainly produced in sebaceous, anal, and salivary glands, and their biological function is unknown. In addition to the lipocalin, albumin, and secretoglobin families, other animal allergens belong to kallikrein, cystatin, or latherin families (Table 1). 

## Component-resolved diagnosis for allergy to furry animals 

In clinical praxis, allergy to animals is diagnosed based on patient’s anamnesis, skin prick testing (SPT), and/or determination of specific IgE antibodies such as by using ImmunoCAP analysis. Both SPT and ImmunoCAP tests are based on natural allergen extracts. However, these extracts are often heterogeneous concerning allergen content, may lack important allergens, and may even contain contaminants which altogether can lead to false-negative or false-positive test result [12]. Although efforts are being made for international standardization of allergy diagnostics and vaccines [61], in reality, usage of poorly standardized extracts severely hampers the correct diagnosis of allergy. Another problem in the correct diagnosis of allergy to animals using allergen extracts is the cross-reactivity within the albumin family (e.g., Fel d 2, Can f 3, and Equ c 3) and within the lipocalin family (e.g., Fel d 4, Can f 6, and Equ c 1). Therefore, the definition of species-specific marker allergens and cross-reactive allergens is important to discriminate between genuine sensitization and cross-sensitization, which is especially important for the correct prescription of allergen-specific immunotherapy (SIT). 

Component-resolved diagnosis (CRD), a new form of allergy diagnosis, can be performed by measuring IgE levels to individual components or by microarray, where more than 100 individual allergens are spotted on one slide [39]. Knowing exact IgE reactivity profiles of the patients is crucial for the selection of patients for appropriate immunotherapy and for monitoring the progress of SIT by measuring allergen-specific IgG antibodies. Several recent studies have shown the usefulness of CRD in allergy to furry animals [4, 7, 13]. The extended microarray based on ISAC technology was used by Curin et al. [13], and it was found that a panel of recombinant and natural cat and dog allergens could efficiently replace the natural allergen extracts regarding sensitivity and specificity, but there is a need to include additional allergen components from horse and small mammals in the microarray. In another study, a microarray was used to explore the pattern of sensitization in two groups of children with allergy to furry animals, the first group with controlled mild-to-moderate asthma and the second group with uncontrolled severe asthma. The authors found that sensitization to the lipocalins seem to be associated with severe asthma in this cohort [37]. The superiority of microarrayed cat and dog allergen molecules over crude animal dander extracts for prediction of cat and dog allergy development from childhood to adolescence was demonstrated in a large cross-sectional and longitudinal population-based study [4]. 

**Table 1.** Animal dander allergens that have been recognized by WHO/IUIS Allergen Nomenclature Sub-Committee (www.allergen.org).

**Allergen**	**MW (kDa)**	**IgE prevalence (%)**	**Cross-reactivity/species specifity**	**Protein family**
Cat (*Felis domesticus*)				
Fel d 1 [44]	18	> 90	Species-specific	Secretoglobin
Fel d 2 [52]	69	14 – 30	Marker for cross-sensitization to other mammalian albumins, responsible for cat-pork syndrome [29, 50, 58]	Serum albumin
Fel d 3 [33]	11	10	nd	Cystatin
Fel d 4 [56]	22	63	Cross-reactive with Can f 6 and Equ c 1; partially cross-reactive with Can f 2 [31, 40, 45]	Lipocalin
Fel d 5 [1]	28, 64	38	Cross-reactive with Fel d 6	Immunoglobulin A
Fel d 6 [2]	28, 94	nd	Cross-reactive with Fel d 5	Immunoglobulin M
Fel d 7 [57]	18	37	Probably cross-reactive with Can f 1	Lipocalin
Fel d 8 [57]	24	19	nd	Latherin
Dog (*Canis familiaris*)				
Can f 1 [35]	18 – 25	49 – 75	Probably cross-reactive with Fel d 7, partially cross-reactive with Can f 2 and human tear lipocalin (TL) with unknown clinical relevance [53]	Lipocalin
Can f 2 [35]	19	10 – 40	Partially cross-reactivity with Can f 1, and Fel d 4 [53, 40]	Lipocalin
Can f 3 [47]	69	16 – 30	Cross-reactive with albumins from other mammals [58]	Serum albumin
Can f 4 [41]	16 – 18	35	Cross-reactive with Bos d 23k [41]	Lipocalin
Can f 5 [42]	28	70	Cross-reactive with human prostate-specific antigen (PSA) [6]	Kallikrein
Can f 6 [31, 45]	27 – 29	38 – 61	Cross-reactive with Fel d 4, Equ c 1 [34]	Lipocalin
Horse (*Equus caballus*)				
Equ c 1 [21]	22 – 25	42 – 76	Cross-reactive with Fel d 4, Can f 6, and Mus m 1 [34, 45, 53]	Lipocalin
Equ c 2 [8]	16	nd	nd	Lipocalin
Equ c 3 [9]	65 – 67	18	Cross-reactive with albumins from other mammals	Serum albumin
Equ c 4 [43]	17	77	nd	Latherin
Rabbit (*Oryctolagus cuniculus*)				
Ory c 1 [5]	17 – 18	nd	nd	Lipocalin
Ory c 2 [5]*	21	nd	nd	Lipocalin
Ory c 3 [27]	19 – 21	77	Species-specific	Secretoglobin
Ory c 4 [28]	24	46	Probably cross-reactive with Fel d 4, Can f 6, and Equ c 1	Lipocalin
Guinea pig (*Cavia porcellus*)				
Cav p 1 [16]	20	70	nd	Lipocalin
Cav p 2 [32]	17	65	Species-specific	Lipocalin
Cav p 3 [32]	18	54	Species-specific	Lipocalin
Cav p 4 [32]	66	52	Cross-reactive with albumins from other mammals	Serum albumin
Cav p 6	18	nd	Probably cross-reactive with Fel d 4, Can f 6, and Equ c 1	Lipocalin
Golden hamster (*Mesocricetus auratus*)				
Mes a 1 [25]	20.5, 24	nd	Probably cross-reactive with European hamster	Lipocalin

MW = molecular weight; nd = not determined; *name used in publications, but not officially recognized.
